# A small contribution on the history of the official implementation of the pediatric otorhinolaryngology in Brazil

**DOI:** 10.1016/j.bjorl.2022.12.005

**Published:** 2023-01-19

**Authors:** Vitor Guo Chen, Shirley Shizue Nagata Pignatari, Reginaldo Raimundo Fujita

**Affiliations:** Disciplina de Otorrinolaringologia Pediátrica, Departamento de Otorrinolaringologia e Cirurgia de Cabeça e Pescoço, Universidade Federal de São Paulo, São Paulo, SP, Brazil

Despite the controversies and even opposition from some colleagues at that time, in 1996, the then called Brazilian Society of Otorhinolaryngology, currently Brazilian Association of Otorhinolaryngology and Cervico-facial Surgery (ABORL-CCF), under the leadership of Professor Doctor Paulo Augusto de Lima Pontes, created the Department of Pediatric Otorhinolaryngology, chaired by Professor Doctor Luc Louis Maurice Weckx and co-chaired by Professor Doctor Eulália Sakano. The main purpose of the newly created department was to advise and encourage the development of ENT research in children and adolescents, spreading knowledge in this area. It started with a very small group, but with a great deal of will and determination.

Concomitantly, in that same year, the Council of the Department of Otorhinolaryngology and Head and Neck Surgery of the Federal University of São Paulo (UNIFESP) requested the university dean, Professor Doctor Hélio Egydio Nogueira, for a Division of Pediatric Otorhinolaryngology. In the Brazilian Federal Register of January 17, 1997, Professor Weckx was appointed to head the new university division. Ref. 1 aimed at assisting exclusively the pediatric population. Since then, a pioneer in Latin America, the Division of Pediatric Otorhinolaryngology at UNIFESP started its fellowship program.

In 1998, thanks to the efforts of Professor Weckx, the newly established Division of Pediatric Otorhinolaryngology at UNIFESP established its first workplace, already equipped for video-nasofibroscopy, audiometry, acoustic rhinomanometry and BERA, allowing quality care and clinical research, and sharing this experience with the academic community. In 2001, through the work of professors Pontes and Weckx, the first Mouth Breathing Center was launched in Brazil as a especial sector of the Pediatric Otorhinolaryngology Division. In a public-private partnership model – Schering Plow® laboratory and the Rotary club – a multidisciplinary and multiprofessional care model was developed especially for children with mouth breathing. Thanks to the work of otorhinolaryngologists, pediatric allergologists, homeopaths and other professionals (dentists, speech therapists and physiotherapists) it was possible to offer comprehensive care to the patient in a single outpatient visit.

In 2004, during the ABORL-CCF general assembly held during the Brazilian Congress of Otorhinolaryngology in Fortaleza-CE, the then Department gained the status of Brazilian Academy of Pediatric Otorhinolaryngology. Professor Weckx was indicated as the first chairman.

Concurrently, the Division of Pediatric Otorhinolaryngology at UNIFESP grew and flourished for 15 years, being brilliantly led also by Professor Weckx until the date of his passing in 2012, after a battle against cancer.^2^ Over the years, new leaderships and new workplaces allowed patients and professionals to have a broader, more modern, and renewed infrastructure. Moreover, the interface with other specialties gained new members, including Pediatric Surgery, Thoracic Surgery and Sleep Medicine. The university Division of Pediatric Otorhinolaryngology maintains the three basic pillars of a university health institution: assistance, teaching and research.

Currently, it is with great pride that we see a well-established and operational Pediatric Otorhinolaryngology throughout Brazil, both in the university and private centers.^3^

## Conflicts of interest

The authors declare no conflicts of interest.
